# Observed Interactions, Challenges, and Opportunities in Student-Led, Web-Based Near-Peer Teaching for Medical Students: Interview Study Among Peer Learners and Peer Teachers

**DOI:** 10.2196/40716

**Published:** 2023-05-15

**Authors:** Evelyn Hui Yi Chan, Vernice Hui Yan Chan, Jannie Roed, Julie Yun Chen

**Affiliations:** 1 Li Ka Shing Faculty of Medicine The University of Hong Kong Hong Kong Hong Kong; 2 Centre for the Enhancement of Teaching and Learning The University of Hong Kong Hong Kong Hong Kong; 3 Department of Family Medicine and Primary Care Li Ka Shing Faculty of Medicine The University of Hong Kong Hong Kong Hong Kong

**Keywords:** peer teaching, peer-assisted learning, medical student, medical education, web-based education, distance learning

## Abstract

**Background:**

Near-peer teaching (NPT) is becoming an increasingly popular pedagogical tool in health professions education. Despite the shift in formal medical education from face-to-face teaching toward encompassing web-based learning activities, NPT has not experienced a similar transition. Apart from the few reports on NPT programs hastily converted to web-based learning in light of the COVID-19 pandemic, no studies to date have explored web-based learning in the specific context of NPT.

**Objective:**

This qualitative study examined the nature of interactions among peer learners (PLs), peer teachers (PTs), and the learning content in a student-led, web-based NPT program for medical students.

**Methods:**

A 5-month-long voluntary NPT program to support first- and second-year medical students’ biomedical science learning in the undergraduate medical curriculum was designed by 2 senior-year medical students and delivered by 25 PTs with 84 PLs participating. In total, 9 PLs and 3 PTs underwent individual semistructured interviews at the end of the program to explore general NPT experience, reasons for joining NPT, the effectiveness of NPT, the demand and importance of NPT, and the feasibility of incorporating NPT in the formal curriculum. Interview transcripts were analyzed using a thematic analysis approach.

**Results:**

The first general theme focused on the nature of student-student, student-teacher, and student-content interactions. Although PLs were engaged in web-based NPT, there was minimal interaction between students, as most PLs preferred to learn passively and remain anonymous. PLs believed the web-based NPT learning process to be a unidirectional transmission of knowledge from teacher to learner, with the teacher responsible for driving the interactions. This was in sharp contrast to PTs’ expectation that both parties shared responsibility for learning in a collaborative effort. The second general theme identified the advantages and disadvantages of delivering NPT on a web platform, which were mainly convenience and teaching skills development and poor interactivity, respectively.

**Conclusions:**

Student-led, web-based NPT offers a flexible and comfortable means of delivering academic and nonacademic guidance to medical students. However, the web-based mode of delivery presents unique challenges in facilitating meaningful interactions among PLs, PTs, and subject content. A blended learning approach may be best suited for this form of student-led NPT program to optimize its efficacy.

## Introduction

### Overview

Medical education has seen a gradual shift toward web-based learning in recent decades [1], even before the COVID-19 pandemic hastened this transition [2]. In contrast, medical education initiatives such as near-peer teaching (NPT) programs have typically been conducted as in-person activities, wherein near peers—students “one or more years senior in training on the same level of the medical education spectrum” [3]—act as peer teachers (PTs) to teach junior students, peer learners (PLs).

Compared with same-level PTs, near peers have a better understanding of the knowledge that students are expected to acquire and potential pitfalls [3]. Meanwhile, they are better equipped to communicate information at an appropriate level and empathize with students than faculty members [3]. Thus, NPT as a pedagogical approach in an increasingly digitalized medical education landscape is an important area of study.

Although there has been extensive literature published on the outcomes of web-based medical education in general [4], the use of web-based means to conduct NPT has been understudied. It was not until the COVID-19 pandemic that NPT programs were forced to take place on the web, leading to several publications commenting on the feasibility, merits, and challenges of delivering NPT on the web to meet the educational demands during the pandemic [5,6]. However, the implementation of NPT under such crisis-ridden circumstances represents “emergency remote teaching,” which should be distinguished from programs intentionally designed to be delivered entirely on the web [7].

This study focused on a voluntary student-designed and student-delivered initiative. To date, to the best of our knowledge, no research has explored in detail medical students’ experiences of student-led NPT purposely delivered on the web.

### Background

The sudden transition of medical education to a web-based setting during the COVID-19 pandemic occurred for both formal teaching and NPT around the globe. Institutions conducted web-based NPT to deliver didactic teaching, clinical clerkships, subinternships, and mentorships during this time [8-11]. Jeong et al [6] developed a web-based peer teaching elective “born of necessity” during the pandemic and found it to be a feasible supplementary learning medium that benefited both PLs and PTs. Meanwhile, Hampshire et al [12] reported that the web-based format of NPT for teaching immunology and microbiology content increased student engagement. Similarly, near-peer surgical teaching for junior doctors using a web-based platform was perceived by trainees as an effective alternative to classroom teaching in terms of overall quality, relevance, and usefulness [13].

However, the emergency adaptation of face-to-face teaching to a web-based mode of delivery faced several challenges. From medical students’ perspectives, barriers to web-based learning include quality assurance of content delivery, educators’ lack of experience in web-based delivery, learners’ acceptance of new learning modalities, and levels of engagement in web-based classes [14]. In a letter to the editor of the journal *Medical Education Online*, Roberts et al [5] reflected on the challenges of restructuring their peer-led teaching sessions into a web-based format during the COVID-19 pandemic. These included maintaining learner engagement, managing learner passivity, and raising the technological skills level of tutors [5].

The limitations of emergency remote teaching and web-based NPT have led to mixed evaluations of their value and efficacy as a pedagogical tool. Although objective outcomes of student performance were equivalent in in-person NPT and web-based NPT established during the pandemic, students perceived web-based NPT of anatomy and radiology to be less effective as a learning tool and felt that PTs were ill-prepared for the small-group sessions [8]. Similarly, students at the University of Malta found web-based, small-group tutorials for anatomy teaching to be ineffective [15]. In contrast, student examination scores, engagement in teaching activities, and evaluations of a web-based pediatric clinical clerkship based on hybrid learning principles and NPT were similar to in-person clerkship outcomes [9].

Optimal strategies to engage students in web-based NPT and student preferences for web-based interaction have not been extensively investigated [16]. Rosenthal et al [16] explored the enjoyment, comfort, engagement, and learning associated with 5 different methods of class participation in a web-based NPT program for emergency medicine developed during the pandemic. They found that calling on students in groups of 3, using web-based group polling software, and asking for volunteer responses in the videoconference platform’s “chat” feature maximized student learning and engagement without compromising enjoyment and comfort. However, the perspectives of PTs were not addressed, which are important in student-led NPT initiatives as the sustainability of such programs relies on participation by the PTs in addition to learners.

No studies have explored the attitudes and perceptions of PLs *and* PTs toward a carefully planned web-based NPT experience. The instructional design and planning process required for effective web-based learning is absent in a majority of emergency remote teaching intended to be a temporary shift of delivery mode during a crisis [7]. Thus, the expectations, experiences, and challenges faced in the implementation of a web-based NPT program *intentionally* designed to be delivered on the web may differ from those reported in the existing literature. This study focuses on the student-student (SS), student-teacher (ST), and student-content (SC) interactions exhibited among PLs and PTs during a student-led, web-based NPT program.

## Methods

### Developing an NPT Initiative at the University of Hong Kong’s Li Ka Shing Faculty of Medicine

The abrupt transition to web-based learning in November 2019, because of social unrest in Hong Kong and the subsequent COVID-19 pandemic, was a challenge for all students but in particular for second-year medical students, as year 2 is recognized as one of the most demanding years of study in the Bachelor of Medicine and Bachelor of Surgery curriculum. Traditionally, senior-year medical students had supported these students on an informal ad hoc basis. However, the NPT program aimed to deliver student-led teaching in a systematic, pedagogically robust manner at this time of need to supplement the formal curriculum by adding value and extending the concepts learned. The fifth-year students who led the NPT initiative collaborated with faculty members to identify the most challenging areas of the year-2 curriculum to identify areas of focus for the NPT sessions. In total, 25 PTs participated in the program, of which 6 (24%) were male and 19 (76%) were female, and 10 (40%) had previous teaching experience (eg, private tutoring). The PTs were provided with a briefing session, a handbook, and optional training opportunities to prepare them for their role. Two training options were co-designed by the student organizers and university staff, namely a course on “Peer-Teaching in Higher Education” delivered by the University of Hong Kong Centre for the Enhancement of Teaching and Learning and a web-based training session on pedagogical approaches and skills for small-group learning run by the Bau Institute of Medical and Health Sciences Education. Both training programs focused on strategies particularly aimed at web-based teaching. Interactive tutorials were held on the web using Zoom (Zoom Video Communications) in small groups of 1 or 2 PTs with 5 to 10 PLs. Each session lasted between 1 and 2 hours.

Throughout the second semester of the 2020 to 2021 academic year, PTs scheduled tutorials on core topics of the year-2 organ system–based preclinical curriculum according to their availability. The tutorial schedule was made available to the year-2 Bachelor of Medicine and Bachelor of Surgery cohort via a social media platform in advance of the sessions and was updated biweekly. Students enrolled in tutorials on a first-come-first-serve rolling basis. Over the 5-month teaching period, 84 PLs participated in the program, of which 38 (45%) were male and 46 (55%) were female. Of these 84 participants, 68 (81%) and 16 (19%) participants were non–degree holders and degree holders, respectively.

### Study Population and Research Questions

A qualitative study was undertaken in which PLs and PTs were identified through purposive sampling to participate in semistructured interviews upon completion of the 5-month-long NPT program. An information sheet and consent form were provided to participants, and 9 PLs and 3 PTs agreed to participate in the study. The overall research questions were as follows: How do students behave in a web-based NPT context? and How does student behavior impact web-based NPT?

### Ethics Approval

Before the data collection, ethics approval was obtained from the University of Hong Kong’s Human Research Ethics Committee (reference EA200224).

### Theoretical Framework

This small study was conducted using semistructured interviews within the research paradigm of narrative inquiry. As stated by Mertova and Webster [17], narrative inquiry is situated within human stories. It is a research method that captures how we as humans experience and perceive events. There is no scientific “validity” attached to the collected data, as there is no attempt to generalize findings. The way we as humans experience a situation is unique to each one of us. Within this paradigm, the researcher investigates experiences of particular events and looks for patterns or themes in the ways participants perceive situations. Through the semistructured interviews, the researchers entered a dialogue with the PLs and PTs to capture their particular experiences in participating in NPT.

### Data Collection

Semistructured interviews were conducted using Zoom and audio recorded. An interview guide was developed for PLs and PTs (Multimedia Appendix 1) to elucidate their thoughts on NPT across five domains as follows: (1) general NPT experience, (2) reasons for joining NPT, (3) the effectiveness of NPT, (4) the demand and importance of NPT, and (5) the feasibility of incorporating NPT in the formal curriculum. Each interview lasted approximately 20 minutes and was transcribed verbatim and anonymized by a third party with no vested interest in the study.

### Data Analysis

Two members of the research team analyzed the transcripts using a thematic analysis approach, which involves 6 phases: familiarization with the data, generating initial codes, searching for themes, reviewing themes, defining and naming themes, and producing the report [18]. They independently applied inductive coding over multiple readings to identify recurrent themes, which were subsequently reviewed and revised, with differences resolved by consensus. Associated quotations were extracted to illustrate the key agreed-upon themes. Once key themes were identified, they were categorized according to the Moore [19] framework of the 3 forms of desirable interactions in distance education (ie, SS, ST, and SC interactions) to examine the nature of PT and PL interactions in web-based NPT and the advantages and disadvantages of web-based NPT.

## Results

### Overview

A total of 9 PLs and 3 PTs were interviewed. Among the 9 PLs, 3 (33%) were male and 6 (67%) were female. Among the 3 PTs, 1 (33%) was male and 2 (67%) were female. The semistructured interviews revealed participants’ perceptions of web-based learning and the nature of the interactions among PLs, PTs, and tutorial content. The themes identified from the thematic analysis are summarized in Textbox 1. Overall, SS interaction was limited in comparison with ST and SC interaction, and PLs and PTs had differing views on what constituted “interaction” in a web-based setting. However, web-based NPT provided a comfortable environment for PLs to learn and PTs to develop their teaching skills.

**Summary of themes (global theme, organizing theme, and basic theme).**

**Types of interactions in web-based near-peer teaching (NPT) [19]**

- Student-student
  Individualistic learning approach
  Students’ perceptions of the level of expertise of their peers and near peers
- Student-teacher
  Preference for anonymity and privacy
  Discrepancy in expectations regarding the roles and responsibilities of peer learners and peer teachers
- Student-content
  Passivity in learning
  Learning priorities
- Advantages and disadvantages of web-based NPT
  Web-based learning environment
  Skills development for web-based teaching

### Nature of Interactions in Web-Based NPT

#### SS Interaction

Both PTs and PLs noticed a lack of SS interaction during the tutorials. Some PLs adopted a passive approach to learning and refrained from speaking aloud or showing their face on camera:

The students weren’t very willing to verbally communicate on Zoom or turn on their cameras or speak to each other.

PT-3

Not all of the students will participate actively in the session, [some] keep muted and keep their camera closed all the time.

PL-2

However, the PLs may not have felt that such a lack of interaction hindered their learning during the session. Most PLs perceived NPT as a means to learn from their near peers solely through the direct transmission of knowledge from those in the senior years rather than an opportunity to collaborate with their immediate peers to develop knowledge together in a collective learning process:

I wouldn’t say that [the lack of student-student interaction] would affect the atmosphere, because I mean we are here to learn, and I [am] just focusing on the tutor but not our classmates. It doesn’t really affect me that much.

PL-4

#### ST Interaction

PTs and PLs generally felt a greater degree of ST interaction than SS interaction, although this was still largely limited to participation by PLs via anonymous platforms or written communication or one-on-one private interactions between the PL and PT:

They [PTs] were kind of making more interaction [with us and would] give us time to ask questions.

PL-6

Especially when we did activities like Kahoot or online games, they [PLs] were very actively participating...I also was pleasantly surprised by how many questions they sent, like a lot of them private messaged me questions about topics they were confused about.

PT-3

Regarding the use of web-based quiz platforms, such as Mentimeter, to promote ST interaction, one of the PLs stated the following:

Most importantly it’s anonymous...so people can’t see who is answering, so I think they will be more brave and interacting. Rather than typing on the chat box on Zoom, I think [it’s] definitely helping [interaction].

PL-5

However, some PLs did not desire or experience much ST interaction in their sessions:

I want to go there and learn so it’s like a peer teacher teach and I listen kind of mode.

PL-1

There is not much interaction. And I think there isn’t much in normal lectures either, so it didn’t really matter because I personally just watch the recorded [lecture] videos...there’s no difference to me.

PL-4

On the other hand, some students preferred more ST interaction than what was available, such as longitudinal interactions spanning beyond the tutorial itself:

The tutor could do more to get to know us and perhaps [offer] some sort of support for our study after the tutorial because...now it’s more like, “Oh, we have like a lecture or a tutorial and then, oh, bye bye.” I think there could be follow-up after the tutorial.

PL-3

#### SC Interaction

The PLs and PTs felt that the PLs were engaged in web-based tutorials, with certain learning activities (such as applying knowledge to clinical scenarios and web-based quizzes) being more effective in encouraging PLs to interact with the subject matter than others. However, the PLs believed that the PT was responsible for driving their engagement with the content instead of it being a self-motivated process:

We tried to come up with a couple of different clinical cases in the form of multiple choice questions to get them to really think about: “Oh, so if you have a patient scenario what might they have? How might you manage them?” A piece of feedback that we received was that the students really enjoyed these kinds of questions and...knowledge synthesis.

PT-1

I always do anticipate that some students will not answer our quizzes [and] not want to participate, so I was really surprised by everyone answering...and all the questions we got really showed that they were listening.

PT-3

Although not many students are participating or actively speaking, but some of the teachers can make us more active in the form of using Menti.

PL-2

PLs had varied opinions toward the modalities used to assess their understanding of the tutorial content. Anonymous quiz platforms were favored over asking and answering questions verbally or in a written format wherein PLs’ identities would be revealed:

Some people are still afraid of having their answers on the chat publicly wrong [but] I think [NPT is] more interactive than the actual lectures.

PL-5

Despite the passivity of certain PLs, their voluntary participation in the extracurricular NPT sessions suggests their engagement with the content:

The schedule of a medical student is really harsh so sometimes one to two hours could be the other time for us to revise. [That was] the first hesitation for us when we first heard of it [NPT], [but after the first session] I think it’s good...that’s why I keep going.

PL-6

#### Conceptualization of Web-Based “Interaction” by PLs and PTs

Although some students hoped to learn passively from the PTs, others recognized the importance of interaction in learning and provided feedback that NPT could be improved by integrating more interactions. However, some PLs viewed “learning” as acquiring strategies for memorizing information rather than attaining a deeper understanding of concepts:

If the session is more interactive it would leave a larger impression like it will help us to better memorize all the stuff mentioned in a session.

PL-2

I [thought] it might be good to attend and see what the seniors do to recite [the cranial nerves], and I think that they really shared some tips that will help me to recite this better.

PL-4

Furthermore, there was a discrepancy in expectations between PLs and PTs regarding the nature of the interaction by PLs during web-based NPT. PTs expressed disappointment in the PLs’ unwillingness to interact through their cameras and microphones:

The first session didn’t really meet my expectations...because I expected them to be even more interactive.

PT-2

It’s quite difficult to teach if they do not open the cameras...I invited them [to] open the camera so it’s more interactive so I can see them nod or see if they understand what I’m talking about.

PT-2

I haven’t really been able to visually see the people I’m kind of tutoring or coaching which is a little bit of a bummer but I completely understand, I mean like 8 or 9pm I don’t really want to be turning my camera on either...without these visual cues, perhaps with their facial expressions or body language, it can be quite difficult to gauge whether we’re getting the point across or whether they find it as engaging as we hope it is...I think some of that feedback would help.

PT-1

Although PLs switched off their cameras and empathized with the increased difficulty this posed to the PTs, they did not believe this hindered interaction overall or their level of engagement and learning in NPT. Evidently, PLs and PTs held contrasting views regarding what constituted student “interaction” in web-based learning, with PLs perceiving a distinction between “interaction” and “engagement” in web-based NPT:

Students don’t like opening their cameras, so the teachers can’t really see us...It affects the teacher more than the student because I guess the student actually is quite interactive in terms of asking questions on the chat or even opening their mic.

PL-5

[The NPT sessions are] quite engaging...so I don’t think that [PLs not turning on their cameras] is a problem.

PL-6

In addition, PLs expected PTs to drive the interactions in web-based NPT:

The tutor could ask us questions and then we’ll answer it.

PL-3

I just wanted them to go through maybe or let me know what the...key points in that topic [are].

PL-5

The senior will demonstrate the correct approach [to the question] and their recommended approach is the key thing to the session.

PL-9

One PT shared the belief and felt it was the role of the teacher to entertain PLs during the session, whereas another PT anticipated NPT sessions to be a shared learning process with equal contribution by PLs and PTs to the discussion:

Before the session, I was really nervous because...I thought it would be quite boring and some students may not like the interaction through Zoom.

PT-2

There is that sort of element of responsibility from the student’s perspective to be responsible and take charge of their own learning.

PT-1

### Advantages of Web-Based NPT

For most PLs, web-based NPT was “flexible” and “convenient” in terms of timing and location as “you can just log on from anywhere” (PL-7). Compared with face-to-face sessions, web-based NPT is more casual and enjoyable, as the web-based format “lessens the stress” (PL-1) and “you can actually enjoy the session more comfortably because you can [be at] home” (PL-6).

PTs concurred with the “time and efficiency” (PT-1) advantages of web-based NPT compared with in-person teaching. They also commented on the more comfortable and safe learning environment on the web, especially with the option of remaining anonymous, which may alleviate PLs’ stress associated with interacting with PTs:

[Students are] a little bit more open to asking questions online...So I think that [teaching online] has made it a little bit more comfortable in terms of creating an open and welcoming learning environment for them...It [also] alleviates some of the pressure and the burden of...if I were to raise my hand up in class and everybody [knows] that it’s me.

PT-1

Online [NPT] makes students who would be shy or unwilling to show up in-person come to a class online because they’re able to turn off their camera and mic and...be as disengaged as they want...I think some people do like to study...in their own environment where they’re comfortable and [have] a choice to be anonymous.

PT-3

With web-based teaching, PTs have fewer logistical concerns and more teaching tools available at their disposal to optimize the learning experience for PLs:

I don’t have to think about...how I’m going to hook up my computer to a projector [because] I can easily do that with screen sharing...so I think the technology is really great and really has allowed us to benefit from online teaching and in fact I think it really works well with this kind of Zoom learning.

PT-1

Moreover, web-based NPT provided PTs with opportunities to practice skills unique to web-based teaching and experience teaching in a virtual setting:

It was very good hands-on to see...how to teach in a Zoom format...It allowed me to kind of see the perspective of our professors.

PT-3

I [applied] some skills that I’ve never thought would be useful, especially through online teaching...to trigger some interest of students and to invite any questions.

PT-1

### Disadvantages of Web-Based NPT

However, conducting web-based NPT has its disadvantages too, particularly in terms of hindering SS and ST interaction:

People are less active when the session is not face-to-face.

PL-1

It’s quite quiet during the Zoom meeting because we don’t want to talk on Zoom and we like to type in the chat...In the future, they can have some face-to-face sessions with the juniors so that the juniors can attend and interact with them.

PL-8

It would have been nice if the group was more interactive with us and with each other...Them interacting with one another [would] probably be easier in-person.

PT-3

## Discussion

### Overview

Although e-learning and NPT separately have become popular pedagogical methods used in the setting of medical education [1,20], there have been few reports on the implementation and outcomes of a student-led NPT program *purposely* designed to be delivered on the web. This qualitative study offers an insight into medical students’ experiences of web-based NPT either as PTs or PLs, in particular, the perceived nature of interactions during tutorials and the advantages and disadvantages of a web-based medium of instruction. Our findings have implications for educators in medicine and other fields seeking to engage students in NPT on web-based platforms by highlighting key considerations, pitfalls, and opportunities for facilitating interactions in web-based NPT.

### Principal Findings

#### Interaction Between Students on the Web

Our study demonstrates that web-based NPT sessions facilitated interactions among PLs, PTs, and subject content to varying degrees. The lack of SS interactions witnessed during NPT was similarly reported in the context of web-based learning by Wut and Xu [21] and Ng [22] among university students and tutors in Hong Kong and by Banna et al [23] in the United States. In addition, Wut and Xu [21] found that web-based classrooms posed challenges to students’ teamwork and group discussion, peer learning through the process of asking questions and formulating solutions, and establishing social presence.

This phenomenon may be explained by the Transactional Distance theory [24], which considers the impact of various types of interaction on the sense of distance a learner feels during web-based learning encounters, and consequently, their engagement and behavior. The absence of face-to-face human contact in web-based settings is likely to increase the transactional distance experienced by the student, thus reducing their sense of belonging and willingness to participate [23].

Other possible factors accounting for limited SS interaction include the unfamiliarity of students with one another, their different learning paces, and the depth of understanding of course material [22]. The students’ personality may also hinder SS interaction. For example, introverted students may prefer to learn on a web-based platform over a face-to-face classroom but enjoy web-based activities that involve working alone rather than in a collaborative manner [25].

However, the importance of SS interaction in learning, such as student satisfaction and performance, is still under debate. Small-group learning has been shown to benefit student achievement considerably more than individual learning [23]. On the other hand, Moore et al [26] reported that most students did not like or want SS interaction in distance education classes, whereas Kuo et al [27] demonstrated that SS interaction was not a substantial predictor of student satisfaction, in contrast to SC and ST interactions. The value and perceived importance of SS interaction in web-based learning may further depend on contextual factors, such as whether collaborative activities or group projects are needed [27].

#### Interaction Between ST and SC

Regarding ST and SC interactions, this study found that PLs mainly adopted a passive learning approach with minimal interaction or interacted with PTs through communication channels that guaranteed anonymity from other PLs. This behavior was surprising to some PTs, who expected PLs to be more open to interacting through their cameras and microphones. Wut and Xu [21] noted similar challenges with university students in Hong Kong being reluctant to openly share their views, ask questions, and request clarifications. Various factors affect students’ reluctance to exchange information in web-based settings. Knowledge-sharing behavior, which may be explicit (eg, sharing documents) or implicit (eg, sharing know-how), has been shown to be lacking in web-based environments compared with face-to-face learning [28]. Using the Transactional Distance theory to understand how web interactions affect knowledge-sharing behavior, Yilmaz [29] reported that higher-quality SS, ST, and SC interactions (among others) reduce individuals’ sense of transactional distance in a web-based environment, which subsequently improves knowledge-sharing behavior.

Culture further impacts knowledge-sharing behavior and one’s predilection for anonymity. In a study of multinational and cross-cultural web-based classes involving students from Hong Kong, Beijing, and the Netherlands [28], the cultural dimensions identified by Hofstede [30] affected knowledge sharing, which included collectivism and individualism (the extent to which individuals in a society are integrated into groups), power distance (the degree of acceptance and expectation of unequal power distribution by less powerful members of society), uncertainty avoidance (how threatened members of a society feel about uncertain or unknown situations), Confucian dynamism (having a long-term or short-term orientation in life), and concern for face (concern over the image of oneself, another party, or both parties). Cultural values may further explain students’ preferences for anonymous peer review, as students from Asian backgrounds (eg, Mainland China, Taiwan, and Hong Kong) are reluctant to criticize their peers’ work to preserve group harmony [31].

In addition, students’ preference to remain passive, private, and anonymous in ST and SC interactions may be related to personality factors, such as being shy or embarrassed to ask questions publicly or being concerned about making mistakes in front of other peers [21]. Alternatively, their behavior may reflect their *surface* approach to learning, aimed at merely reproducing learning material in the absence of reflection about the purpose of knowing the information or formation of connections between the information [32]. This was apparent in 4 PLs who expected NPT to be an act of the PT transmitting knowledge in a unidirectional manner to the PLs who received it passively and “learning” to occur from rote memorization of facts rather than understanding the information. In contrast, PTs unanimously adopted a “deep” learning approach by finding patterns in the knowledge and explaining the principles underpinning information, which they anticipated PLs would emulate but did not in practice. Mirghani et al [32] similarly reported that first- and second-year medical students preferred a “surface” learning approach, whereas senior-year students were more likely to adopt a “deep” learning approach. Considering that the learning environment and culture plays a role in shaping students’ learning approach, this finding is not surprising because the heavy workload and examination-based assessment of preclinical medical education makes “deep” learning difficult for students [32]. Nevertheless, PLs’ passive “surface” learning approach has implications for the academic outcomes of web-based NPT, as this approach is associated with poor academic performance [33,34].

However, although extensive ST and SC interactions by active students who reveal their identities represent tangible indicators of the individual’s engagement and are assumed to enhance learning, anonymous interactions or the absence of visible activity do not equate to disinterest or disengagement with web-based learning [35]. “Lurkers” who are present but remain inactive in web-based discourse with their peers and instructors are nevertheless still learning, despite not visibly participating [36,37]. Furthermore, there is no substantial difference between active and passive activities on student engagement levels in web-based courses, although active means of interaction may offer additional benefits, such as strengthening students’ social presence and potentially reducing social isolation [38]. This may be an important consideration for NPT programs that aim to offer social support, in addition to academic guidance.

#### Advantages and Disadvantages of Student-Led, Web-Based NPT

Implementing a student-led NPT program using a web-based platform has its unique advantages and disadvantages. Flexibility and comfort level are commonly cited strengths of web-based education, especially in uncertain circumstances such as during the COVID-19 pandemic [39,40]. Besides tutoring, web-based, student-led NPT platforms may also be used to provide psychological support and nonacademic advice [41,42]. However, web-based environments may still be less conducive to sharing socioemotional information than in person [43]. In addition, web-based NPT develops the teaching skills and technological literacy of PTs, which are essential professional competencies in the modern era of medicine, given the likelihood for web-based learning pedagogy to persist in the future [21,44].

### Comparison With Prior Work: Outcomes of Student-Led, Web-Based Learning and Face-to-Face Learning

Evaluation of learning should encompass not only the extent of information acquired by students but also the social interaction and “connectedness” that students feel throughout the process. As Gilbert and Moore [45] emphasized, there is a need to assess both “informational/instructional interactivity” and “social/organizational interactivity” when comparing traditional and web-based instruction. Future research should compare web-based and face-to-face delivery of NPT with regard to the academic and nonacademic facets of students’ learning experiences. On the other hand, a study conducted by Foo et al [2] on medical students from the same institution as this study found that students performed significantly worse in problem-based learning tutorials conducted on the web than in person from the perspective of the tutors, specifically in the domains of participation, communication, preparation, critical thinking, group skills, and total score. More research is needed with regard to student performance in the context of NPT and with students’ perceptions (such as satisfaction) taken into consideration.

Specific aspects of the learning experience that are better supported by web-based or in-person interactions should be clarified. Paechter and Maier [43] highlighted that the students undertaking courses at Austrian universities had clear preferences for web-based or face-to-face learning depending on the particular learning objective or learning process. Students favored web-based communication for SS interactions that merely involved the dissemination of information to peers but face-to-face communication in situations that required higher cognitive presence (such as cooperative learning, agreeing on a shared meaning with other learners, or reaching a joint solution) [43]. For ST interactions, web-based communication was deemed more appropriate for the rapid exchange of information with tutors (such as receiving feedback), whereas face-to-face interaction was preferred in situations in which tutors developed the knowledge of students (eg, by facilitating the acquisition of knowledge) [43]. To establish positive SS and ST social relations, students advocated for face-to-face interaction [43]. It is uncertain whether such appraisals of preferred interactions are applicable to informal NPT settings dominated by synchronous learning activities. Research focusing on students’ preferences for specific aspects of NPT in the context of medical education is necessary.

### Future Directions

Moving forward, student-coordinated NPT programs for medical students in Hong Kong should be delivered in a manner that balances convenience and flexibility without compromising social and organizational interactivity, informational and instructional interactivity, and program sustainability [45]. Considering the results of this study and students’ preferences for web-based or face-to-face interaction, depending on the learning objective [43], NPT may benefit from a blended learning approach that incorporates traditional face-to-face learning and e-learning.

Blended learning is already widely implemented in formal medical education, with meta-analyses demonstrating significantly improved knowledge acquisition and outcomes compared with traditional learning in health education [46,47]. It may similarly be optimal to conduct student-led NPT tutorials using this method, as the factors restricting SS, ST, and SC interactions in NPT, as identified by this study, are likely explained by the limited social and organizational interactivity offered on the web compared with in-person interaction, resulting in students’ heightened sense of transactional distance, unfamiliarity with their peers, lack of belonging, and reluctance to actively participate [23]. NPT in particular is heavily centered on collaborative learning and has the secondary aim of developing students’ social support network; thus, face-to-face elements are suggested and preferred by students to facilitate their cooperation on tasks and share socioemotional information [43], improve social presence and relations, and reduce social isolation [38].

In addition, the web-based component of student-led NPT should be retained as its convenience, efficiency, and comfort level reduce students’ barriers to participation as a PL or PT, hence ensuring the sustainability of the program. e-Learning for health professions is associated with equivalent or even superior outcomes than traditional learning in terms of knowledge, skills, attitudes, and satisfaction [4]; hence, the quality of learning in NPT should not be inferior to face-to-face learning if conducted on the web. Strategies such as creating anonymous quizzes [35] or assigning roles to each student in small-group discussions [48] can maintain student engagement on the web. PTs should be trained in such web-based teaching strategies to facilitate interactions that enable effective learning and standardize the quality of teaching. PTs should remind PLs of their shared responsibility for learning and their expected active contribution to tutorials [23]. Moreover, the web-based elements of NPT can be extended beyond the delivery of tutorials. Asynchronous measures such as a web-based discussion forum or a group created on social networking sites can promote interaction, collaboration, active participation, the sharing of knowledge and resources, and critical thinking [23]. PLs can be assigned to groups that are kept the same throughout their study to strengthen group cohesiveness and longitudinal relationships.

### Limitations

First, a limitation of this study is the lack of a comparison group of students participating in face-to-face NPT and comparison of web-based NPT with web-based classes that are a part of the formal curriculum. Consequently, the findings regarding the nature and outcomes of SS, ST, and SC interactions may not be a result of the web-based format of NPT alone but rather influenced by other elements of NPT implementation, such as group size, educational distance between PTs and PLs, teaching skills of PTs, or the students’ personalities. Future studies should compare interactions among students in web-based and face-to-face NPT, exploring in further detail the specific aspects of near-peer education that benefit most with either the mode of instruction and the underlying causes of the learning behaviors that shape interaction. Second, although the qualitative research methodology used enables a detailed understanding of participants’ perceptions and feelings about NPT to inform student-centered pedagogical design, it does not allow for an objective assessment of learning outcomes for PLs (such as academic performance, satisfaction, and engagement) and PTs (such as teaching competencies and academic performance). A quantitative study with a larger sample size would allow such outcomes to be explored to guide future NPT programs.

### Conclusions

This study reveals the nature of the SS, ST, and SC interactions that take place in student-led NPT tutorials conducted on the web for medical students, designed and delivered by medical students. Despite the web-based learning environment being convenient and comfortable, students refrained from participating in active and collaborative ways. Nevertheless, web-based NPT can serve as a useful supplement to formal medical education by providing an easily accessible platform for PLs to receive academic and psychosocial support and for PTs to develop their competencies as educators in a digital era. Future directions of NPT should make use of the strengths of both web-based and face-to-face modalities to foster meaningful interactions and maximize learning, whereas further research should explore the subjective experience and objective outcomes of web-based versus face-to-face NPT.

## Appendix

Multimedia Appendix 1 Interview guide for peer learners and peer teachers.

## Abbreviations

NPT: near-peer teaching
PL: peer learner
PT: peer teacher
SC: student-content
SS: student-student
ST: student-teacher

## Data Availability

The data sets generated during and analyzed during this study are available from the corresponding author upon reasonable request.
