# Supplementary material for: Observed Interactions, Challenges, and Opportunities in Student-Led, Web-Based Near-Peer Teaching for Medical Students: Interview Study Among Peer Learners and Peer Teachers
Source: JMIR Med Educ. 2023 May 15;9:e40716. doi: 10.2196/40716 (PMC10227701; doi:10.2196/40716)
Supplement: Multimedia Appendix 1 [file mededu_v9i1e40716_app1.pdf]

Multimedia Appendix 1: Interview guide for peer learners and peer teachers

Peer Learners:

<u>General NPT experience</u>

1. How did you find the NPT sessions? What went well and what didn't? And why?
2. What do you think about the online format of the NPT sessions? Can you describe 2 advantages or disadvantages?

<u>Reasons for joining NPT</u>

3. Why did you join NPT?
4. What hesitations did you have about joining NPT? How has your experience changed your perception (or not), and why?
5. What do you think hinders students from joining the NPT program as a PL?

<u>Effectiveness of NPT</u>

6. What were your expectations of NPT? In what way did the NPT sessions meet your expectations (or not)?
7. How did you benefit (or not) from the NPT sessions? What did you gain (if anything) out of the NPT sessions that you would not be able to gain from the formal curriculum?
8. In your view, what are the benefits of having a senior student teach you instead of a lecturer? What are the disadvantages?
9. What interactions / learning processes took place during NPT sessions which were helpful to you? Can you recall an example and explain how it was helpful? Anything that wasn't effective, and why?

<u>Demand/Importance of NPT</u>

10. Why do you think it is important (or not important) to have an NPT program for medical students?
11. Would you like to be a peer teacher as you progress to more senior years? Why or why not? What benefits do you think you will get as a peer teacher? What sacrifices do you think you will need to make as a peer teacher?

<u>Feasibility of incorporating NPT in the formal curriculum</u>

12. What are your thoughts about peer teaching sessions being part of the formal curriculum, and why?
    [i.e. More advanced students would teach additional sessions as part of the formal course, and the students teaching would gain credits for their contributions]

13. *(Follow-up to Q14 if student thinks NPT should be part of the formal curriculum)*
    a. How can peer teachers help in the formal curriculum *[e.g. think about your experience in various teaching blocks, PBL, labs, practicals, longitudinal programs (MH, HRP, PCP, etc.)]*?
    b. What do you think are the barriers to implementing your suggestions?

14. What other support and resources would help to enhance the NPT experience?

Peer Teachers:

General NPT experience

1. How did you find the NPT sessions? What went well and what didn't? And why?
2. How did you find the teaching skills training opportunities (CETL course and/or BIMHSE sharing session)? Can you mention two things from the training that was useful in your teaching? Anything missing?

3. What do you think about the online format of the NPT sessions? Can you describe 2 advantages or disadvantages?

Reasons for joining NPT

4. Why did you join NPT?

5. What hesitations did you have about joining NPT? How has your experience changed your perception (or not), and why?
6. What do you think hinders students from joining the NPT program as a PT?

Effectiveness of NPT

7. What were your expectations of NPT? In what way did the NPT sessions meet your expectations (or not)?
8. How did you benefit (or not) from the NPT sessions? What did you gain (if anything) out of the NPT sessions that you would not be able to gain from the formal curriculum?

Demand/Importance of NPT

9. Why do you think it is important (or not important) to have an NPT program for medical students?
10. What benefits did you get being a peer teacher? What sacrifices did you make being a peer teacher? Would you recommend others to become peer teachers, and why?

Feasibility of incorporating NPT in the formal curriculum

11. What are your thoughts about peer teaching sessions being part of the formal curriculum, and why?
    [i.e. More advanced students would teach additional sessions as part of the formal course, and the students teaching would gain credits for their contributions]

12. *(Follow-up to Q14 if student thinks NPT should be part of the formal curriculum)*
    a. How can peer teachers help in the formal curriculum *[e.g. think about your experience in various teaching blocks, PBL, labs, practicals, longitudinal programs (MH, HRP, PCP, etc.)]*?
    b. What do you think are the barriers to implementing your suggestions?

13. What other support and resources would help to enhance the NPT experience?

This is a Multimedia Appendix to a full manuscript published in the J Med Internet Res.
http://dx.doi.org/10.2196/jmir.40176
